# Low Gamma-Glutamyl Transferase Cholestasis in a Patient With X-Linked Myotubular Myopathy and Crohn's Disease

**DOI:** 10.14309/crj.0000000000001240

**Published:** 2024-01-22

**Authors:** Rasha Abi Radi Abou Jaoudeh, Brendan McCleary, Kadakkal Radhakrishnan

**Affiliations:** 1 Division of Pediatrics and Adolescent Medicine, Cleveland Clinic Children's Center, Cleveland, OH; 2 Division of Diagnostic Radiology, Cleveland Clinic Children's Center, Cleveland, OH; 3 Division of Pediatric Gastroenterology, Hepatology and Nutrition, Cleveland Clinic Children's Center, Cleveland, OH

**Keywords:** X-linked myotubular myopathy, cholestasis, primary sclerosing cholangitis, progressive familial intrahepatic cholestasis, Crohn's disease

## Abstract

X-linked myotubular myopathy (XLMTM) is a neuromuscular disorder manifesting at birth with hypotonia and respiratory distress. We describe the XLMTM case presenting at birth who developed normal gamma-glutamyl transferase cholestasis at 1 year of age. He was also diagnosed with Crohn's disease 4 years later. His cholestasis could be attributed to progressive familial intrahepatic cholestasis (PFIC) or primary sclerosing cholangitis in the setting of Crohn's disease. However, genetic testing ruled-out PFIC, and his radiographic and liver biopsy findings were not suggestive of primary sclerosing cholangitis. We believe that this cholestasis is related to XLMTM leading to a PFIC-like state.

## INTRODUCTION

X-linked myotubular myopathy (XLMTM) is a neuromuscular disorder that manifests at birth with hypotonia and respiratory distress. We present the XLMTM case who developed cholestasis with normal gamma-glutamyl transferase (GGT) at 1 year of age and was then diagnosed with Crohn's disease 4 years later. We propose that his cholestasis is related to XLMTM leading to a progressive familial intrahepatic cholestasis (PFIC)-like state (1–4) rather than primary sclerosing cholangitis (PSC) or PFIC.

## CASE REPORT

The patient was a boy born at 36 + 7 weeks of gestation to a 34-year-old mother by spontaneous vaginal delivery. The pregnancy was uncomplicated, but the patient was born with profound hypotonia and respiratory failure. Head ultrasound, brain magnetic resonance imaging and electroencephalogram were normal. Metabolic testing including long chain fatty acids, acylcarnitine profile, copper, ceruloplasmin, myasthenia gravis antibodies, cerebrospinal fluid amino acids and lactic acid, Prader Willi methylation, spinal muscular atrophy DNA study, and singe nucleotide polymorphism array were all unremarkable.

Electromyography showed no evidence of chronic motor axon loss, myopathy, or neuromuscular junction dysfunction. Muscle biopsy was suggestive of centronuclear vs myotubular myopathy. Further work-up with electron transport chain complexes demonstrated normal activity in fresh skeletal muscle and mitochondria. Whole-exome sequencing showed X-linked centronuclear myopathy-hemizygous c1262G>A (p.R421Q) mutation in the myotubularin (*MTM1*) gene. Family history was only significant for a maternal third cousin born hypotonic.

**Figure 1.**
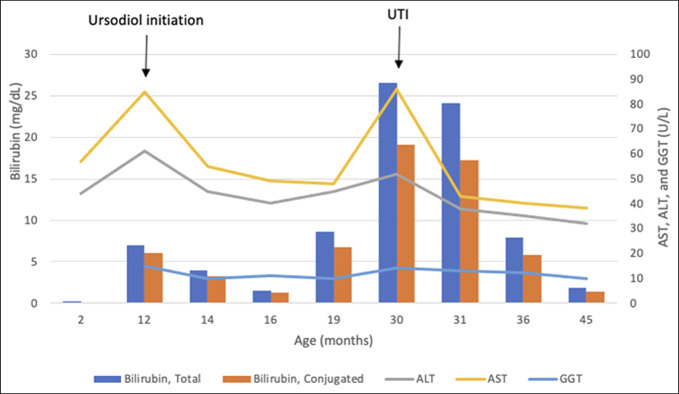
Time course of laboratory findings and selected clinical events. ALT, alanine aminotransferase; AST, aspartate aminotransferase; GGT, gamma-glutamyl transferase; UTI, urinary tract infection.

At 1 year of age, the patient presented with new onset jaundice. His total bilirubin was 7.0 mg/dL (0.0–1.5 mg/dL), conjugated bilirubin 6.1 mg/dL (0.0–0.4 mg/dL), alkaline phosphatase 354 U/L (80–350 U/L), aspartate aminotransferase 86 (10–60 U/L), GGT 15 U/L (0–50 U/L), and alanine aminotransferase 61 U/L (5–50 U/L) (Figure 1). Liver ultrasound showed a homogenous liver without evidence of a mass or ductal dilatation. The gallbladder was thin-walled with echogenic debris. There was no pancreatic or common bile duct dilatation. His conjugated hyperbilirubinemia and increase in transaminases were attributed to microsludge, and he was started on ursodiol. After 2 months of treatment, his cholestasis improved (conjugated bilirubin 1.3 mg/dL) (Figure 1). However, 5 months later, he developed pruritus and his conjugated bilirubin and bile acids increased and continued to trend upward suggesting that he had significant cholestasis. His whole-exome sequencing was reanalyzed, and no known genetic mutations to account for cholestasis were found including PFIC.

**Figure 2.**
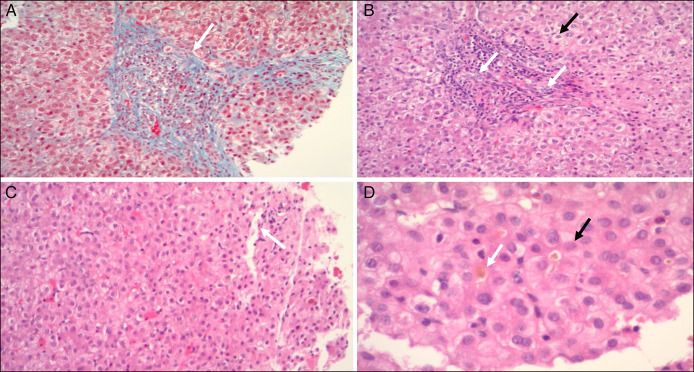
Liver biopsy pathology images showing stellate portal fibrosis (white arrow) (trichrome stain, 20× field) (A), portal inflammation with ductular proliferation (white arrow) and hepatocyte ballooning (black arrow) (H&E, 20× field) (B), hepatic lobular disarray with normal central vein (white arrow) (H&E, 20× field) (C), and canalicular cholestasis (white arrow) with rosette formation (black arrow) (H&E, 60× field) (D). H&E, hematoxylin and eosin.

The patient underwent an uncomplicated cholecystectomy at 30 months of age, after being found to have worsening cholestasis and biliary sludge on ultrasound in the setting of Enterococcus and *Klebsiella pneumoniae* urinary tract infection (Figure 1). During this time, he remained on high-dose ursodiol (125 mg 3 times a day). After resolution of the infection, bilirubin levels improved. His liver biopsy (Figure 2) showed mild hepatic portal inflammation (lymphocytes and neutrophils), stellate portal fibrosis with trichrome stain, and mild ductular proliferation with marked canalicular cholestasis. Otherwise, the liver morphology was within normal limits with unremarkable liver vascular structures and no suggestion of bile duct inflammation or injury.

At 5 years of age, the patient developed nonbloody diarrhea that persisted for 6 months. Endoscopy showed visually normal esophageal, gastric, and duodenal mucosa with scattered ulcerations in the rectum and sigmoid, and surgical pathology showed chronic gastritis with nonnecrotizing granuloma formation and focal active colitis involving the rectum. These findings were concerning for Crohn's disease. After discussing all available treatment options, parents opted for acetylsalicylic acid which resulted in a rash and was thus stopped. Subsequently, the patient was started on short course of prednisone therapy with improvement in diarrhea. At this time, his parents opted for exclusive enteral nutrition and he was successfully weaned off of prednisone. He continued to demonstrate normal GGT cholestasis, with some increase in transaminases and with bile acids trending between 135 and 225 μmol/L over the years. Repeat magnetic resonance cholangiopancreatography showed stable biliary system and no suggestion of PSC. The patient subsequently was started on adalimumab for Crohn's disease for worsening symptoms after repeat upper and lower endoscopy with good symptomatic improvement.

## DISCUSSION

XLMTM is a rare (1 in 50,000 male births)^5^ neuromuscular disease caused by a mutation in the *MTM1* gene. Most patients with XLMTM present at birth with respiratory distress and hypotonia,^6^ as is the case with our patient.^1^ Given that MTM1 is a ubiquitous protein, this disease can affect several organs other than skeletal muscles.^7^

Studies have shown that hepatobiliary disease in patients with XLMTM is independent of the age of the patient or severity of the disease.^1–4^ D'Amico et al^2^ studied 12 patients with XLMTM for 10 years, and they found that 5 of 12 had elevated transaminases and 7 of 12 had abnormal hepatic echogenicity. Only 1 of 12 had 3 pruritic cholestatic jaundice episodes, and similar to our patient, some of these episodes occurred after exposure to stressors such as infections and were characterized by conjugated hyperbilirubinemia and increase in transaminases and bile acids, while GGT remained normal. In a case series by Molera et al,^1^ 5 patients with XLMTM and consequent intrahepatic cholestasis were studied and they all showed recurring elevations in serum bile acids and hyperbilirubinemia with normal GGT levels. Similar to our patient, intercurrent illnesses or stressors led to exacerbation in cholestasis (Figure 1). All but 1 patient responded to choleretic treatment. This patient's liver biopsy showed considerable intrahepatic cholestasis with canalicular bile plugging, giant cell transformation, and portal field fibrosis, and he eventually progressed to end-stage liver failure.

MTM1 is a lipid phosphatase that facilitates hepatic/biliary exocytosis by hydrolyzing phosphatidylinositol 3-monophosphate,^8^ and allowing recycling of endosomes to the cell surface and the formation of autophagosomes. In a preclinical trial by Karolczak et al,^9^ loss-of-function mutation in MTM1 was shown to disrupt the structure of bile canaliculi and the trafficking through them which led to impaired in bile flux. Hence, in XLMTM, the deficient MTM1 function disrupts phosphatidylinositol 3-monophosphate homeostasis and impairs the transport of bile acids,^10^ which ultimately leads to hepatobiliary disease.

**Figure 3.**
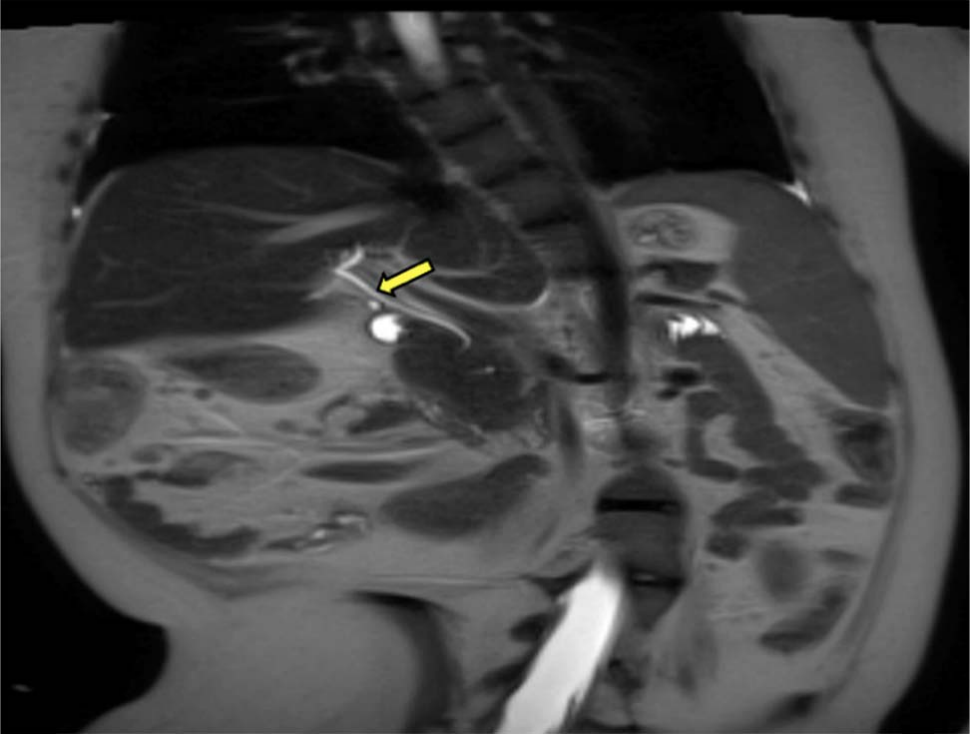
HASTE MRI showing a smooth, nondilated common bile duct (yellow arrow). HASTE, half Fourier single-shot turbo spin-echo; MRI, magnetic resonance image.

Our patient developed Crohn's disease later on in life, and it was therefore important to rule out PSC as a cause for his cholestasis, given that these entities are highly correlated. However, the absence of derangement in the intrahepatic and extrahepatic bile ducts on magnetic resonance imaging (Figure 3) along with the normal GGT and the lack of PSC features on the liver biopsy (Figure 2) made this diagnosis less likely. In addition, autoimmune liver disease was ruled out by biopsy (Figure 2) and negative antibody panel.

PFIC is another entity that can present with jaundice and pruritis that was considered in our patient given the normal GGT.^11^ The liver biopsy (Figure 2) showed mild ductular proliferation with canalicular cholestasis which is seen in PFIC1 and 2.^12^ However, genetic testing for PFIC in our patient was negative. This all points out that our patient was experiencing a PFIC-like state most likely as a result of his underlying XLMTM.

## DISCLOSURES

Author contributions: R. Jaoudeh and K. Radhakrishnan were responsible for manuscript concept and design. All authors were involved in drafting and critical revision of the manuscript and final approval and agreement to be accountable for all aspects of the version to be published. K. Radhakrishnan is the article guarantor.

Financial disclosure: None to report.

Informed consent was obtained for this case report.
